# Identification of uveitis-associated functions based on the feature selection analysis of gene ontology and Kyoto Encyclopedia of Genes and Genomes pathway enrichment scores

**DOI:** 10.3389/fnmol.2022.1007352

**Published:** 2022-09-08

**Authors:** Shiheng Lu, Hui Wang, Jian Zhang

**Affiliations:** ^1^Department of Ophthalmology, Shanghai Eye Disease Prevention and Treatment Center, Shanghai Eye Hospital, Shanghai, China; ^2^Department of Ophthalmology, Shanghai General Hospital, Shanghai Jiao Tong University School of Medicine, Shanghai, China; ^3^Shanghai Key Laboratory of Ocular Fundus Diseases, Shanghai, China; ^4^Shanghai Engineering Center for Visual Science and Photomedicine, Shanghai, China; ^5^National Clinical Research Center for Eye Diseases, Shanghai, China; ^6^Shanghai Engineering Research Center for Precise Diagnosis and Treatment of Eye Diseases, Shanghai, China; ^7^Department of Orthopedics, Shanghai Yangpu Hospital of Traditional Chinese Medicine, Shanghai, China

**Keywords:** uveitis, enrichment theory, gene ontology, KEGG pathway, feature selection

## Abstract

Uveitis is a typical type of eye inflammation affecting the middle layer of eye (i.e., uvea layer) and can lead to blindness in middle-aged and young people. Therefore, a comprehensive study determining the disease susceptibility and the underlying mechanisms for uveitis initiation and progression is urgently needed for the development of effective treatments. In the present study, 108 uveitis-related genes are collected on the basis of literature mining, and 17,560 other human genes are collected from the Ensembl database, which are treated as non-uveitis genes. Uveitis- and non-uveitis-related genes are then encoded by gene ontology (GO) and Kyoto Encyclopedia of Genes and Genomes (KEGG) enrichment scores based on the genes and their neighbors in STRING, resulting in 20,681 GO term features and 297 KEGG pathway features. Subsequently, we identify functions and biological processes that can distinguish uveitis-related genes from other human genes by using an integrated feature selection method, which incorporate feature filtering method (Boruta) and four feature importance assessment methods (i.e., LASSO, LightGBM, MCFS, and mRMR). Some essential GO terms and KEGG pathways related to uveitis, such as GO:0001841 (neural tube formation), has04612 (antigen processing and presentation in human beings), and GO:0043379 (memory T cell differentiation), are identified. The plausibility of the association of mined functional features with uveitis is verified on the basis of the literature. Overall, several advanced machine learning methods are used in the current study to uncover specific functions of uveitis and provide a theoretical foundation for the clinical treatment of uveitis.

## Introduction

Uveitis is a typical type of eye inflammation affecting the middle layer of eye, i.e., the uvea layer (Chang et al., 2006; Mutawa and Alzuwawi, 2019). Patients with uveitis have the following characteristics: various vision-associated symptoms, including but not restricted to light sensitivity and vision field floating spots (Selmi, 2014), and eye tissue-associated symptoms, including eye redness and pain (Mandeville et al., 2001). Different from other eye-associated diseases (like age-related macular degeneration), uveitis has an acute type, which initiates and progresses quite fast into the chronic type (Chen et al., 2015). Acute uveitis can cause severe symptoms, like blindness, in less than six weeks. In 2018, more than 30,000 new cases of blindness are confirmed to be caused by uveitis, which has been identified as one of the main reasons for blindness (Suttorp-Schulten and Rothova, 1996; Durrani et al., 2004). Thus, uveitis is a key threat for human eye health that cannot be ignored.

The pathogenesis of uveitis has been partially revealed. Uveitis can be easily divided into infectious and noninfectious uveitis in accordance with the cause of uveitis initiation (Takase et al., 2006). For infectious uveitis, virus [like HSV, herpes simplex virus (Wensing et al., 2018), varicella zoster virus (Kido et al., 2008), and cytomegalovirus (Chee and Jap, 2010)], and bacteria [like mycobacteria (Rao et al., 2006)] have all been reported to participate in the pathogenesis of uveitis. For noninfectious uveitis, multiple systematic immune diseases, including Behcet’s disease, sarcoidosis, Vogt–Koyanagi–Harada disease, have also been reported to be associated with uveitis initiation and progression (Takeuchi et al., 2021). Although multiple pathogenetic factors have been validated to be associated with uveitis, the disease susceptibility and underlying mechanisms for uveitis initiation and progression have not been systematically summarized and clarified.

Gene ontology (GO) is an integrated bioinformatics initiative for computational analyses on the biological process, cellular component, and molecular function across different species (Consortium, 2019). Kyoto Encyclopedia of Genes and Genomes (KEGG) is another bioinformatics tool that describes the networks of genes and molecules (Kanehisa and Goto, 2000). Each gene can be encoded into a vector by extracting enrichment scores of the gene set, including itself and its immediate neighbors in STRING, and GO terms or KEGG pathways. A high enrichment score for the gene and one GO term or KEGG pathway indicates a close relationship. In this study, we try to attribute the initiation and progression of uveitis to specific biological functions described by GO terms and KEGG pathways, providing a new computational analysis to explore the pathogenesis of this complex disease. To identify key biological functions associated with uveitis, multiple machine learning algorithms, including least absolute shrinkage and selection operator (LASSO) (Tibshirani, 1996), light gradient boosting machine (LightGBM) (Ke et al., 2017), Monte Carlo feature selection (MCFS) (Micha et al., 2008), and minimum redundancy maximum relevance (mRMR) (Peng et al., 2005) are introduced, which are widely used for disease pathogenic factor recognition in previous publications.

As described above, we apply multiple machine learning algorithms to recognize key GO terms and KEGG pathways that can describe uveitis pathogenesis. The comparison between the results yielded by different machine learning algorithms can help us fully identify key functions that contribute to uveitis pathogenesis. The biological functions associated with uveitis found by this research are backed up by the literature, validating the efficacy and accuracy of machine learning-based gene function analysis and pathogenesis exploration.

## Materials and methods

### Data acquisition

Literature mining in PubMed^1^ is used to collect uveitis-related genes. In previous studies, Lu et al. (2017, 2018) conducted a PubMed search by using the keywords “uveitis” and obtained 744 relevant articles. A total of 98 review publications with basic summary of uveitis-related genes are manually reviewed. A total of 121 genes are identified from 96 out of 98 review papers reporting functional genes that may be significant for uveitis pathogenesis. Among these genes, 108 are annotated by GO terms and KEGG pathways, and those without annotation information are removed. These 108 genes obtained are used as positive samples. A total of 17,560 other human genes containing GO and KEGG annotation information are collected from Ensembl database and used as negative samples.

### Feature construction

Uveitis- and non-uveitis-related genes should be transformed into some features so that they can be processed by generally machine learning algorithms (Zhou et al., 2020; Li et al., 2022; Pan et al., 2022; Tang and Chen, 2022; Wang and Chen, 2022; Wu and Chen, 2022; Yang and Chen, 2022). Here, we use GO and KEGG enrichment theory to generate numerical values for representing each gene (Carmona-Saez et al., 2007; Huang et al., 2011; Huang et al., 2012).

Gene ontology enrichment indicates the association between GO terms and genes. For one gene *g*, its direct neighbors in STRING and *g* are termed as *neighbors*_*g*_ genes in this paper. A score is calculated for *neighbors*_*g*_ genes and each GO term *GO*_*j*_, which is commonly referred to as the GO enrichment score. The score is computed as the −log_10_ of the hypergeometric *P*-value for *neighbors*_*g*_ genes and the gene set, in which genes are annotated by the *GO*_*j*_ (called *GO*_*j*_ gene set). The equation is shown as follows:


(1) ESGO(neighborsg,GOj)=-log10⁡(∑x=kn(mx)(N-mn-x)(Nn)),

where *k* is the number of *neighbors*_*g*_ genes that appear in the *GO*_*j*_ gene set, *m* is the number of genes in the *GO*_*j*_ gene set, *n* is the number of *neighbors*_*g*_ genes, and *N* is the total number of genes considered (gene universe). A high score for *neighbors*_*g*_ genes and *GO*_*j*_ indicates the close relationship between *g* and *GO*_*j*_. Finally, 20,681 GO term enrichment features are obtained.

Similarly, for *neighbors*_*g*_ genes and each KEGG pathway *KEGG*_*j*_, the enrichment score is calculated to evaluate the relationship between *g* and *KEGG*_*j*_ by using the same method described above. The equation is shown as follows:


(2) ESKEGG(neighborsg,KEGGj)=-log10⁡(∑x=kn(mx)(N-mn-x)(Nn)),

where *k* is the number of *neighbors*_*g*_ genes that are also in the *KEGG*_*j*_ gene set, *m* is the number of genes in the *KEGG*_*j*_ gene set, *n* is the number of *neighbors*_*g*_ genes, and *N* is the total number of genes considered (gene universe). A high score indicates the close relationship between *g* and *KEGG*_*j*_. Finally, 297 KEGG pathway enrichment features are obtained.

In summary, a gene *g* can be presented as a vector *v*(*g*) consisting of 20,681 GO term features and 297 KEGG pathway features. *v*(*g*) is presented by


(3) $$v\,\left(g\right)=\begin{array}{ccc} (ES_{G\,O}\left(neighbors_{g},GO_{1}\right),\ldots ,ES_{G\,O} & & \\ \left(n\,e\,i\,g\,h\,b\,o\,r\,s_{g},G\,O_{20681}\right), & & \\ E\,S_{K\,E\,G\,G}\,\left(n\,e\,i\,g\,h\,b\,o\,r\,s_{g},K\,E\,G\,G_{1}\right),\ldots ,E\,S_{K\,E\,G\,G} & & \\ \left(neighbors_{g},KEGG_{297}\right)){}^{T} & & \end{array}.$$

Combining the 17,668 samples (108 positive samples and 17,560 negative samples), a two-dimensional matrix is obtained for subsequent analysis.

### Feature filtering with Boruta

Lots of GO terms and KEGG pathways are involved in this analysis. Evidently, only a few of them are highly related to uveitis, which can be extracted by advanced machine learning algorithms. Here, the Boruta method is first applied to exclude irrelevant GO terms and KEGG pathways and keep essential ones.

Boruta is a widely used feature filtering method that relies on two concepts, i.e., shadow features and binomial distribution (Carmona-Saez et al., 2007; Kursa and Rudnicki, 2010; Huang et al., 2022; Zhou et al., 2022). Features in Boruta compete with their random versions, which are created by random shuffling. In terms of feature importance, a feature is called “important” if its importance is superior to its random counterpart. In each run, Boruta keeps “important” features. The second strategy, binomial distribution, focuses on obtaining features with the probability of being kept in all runs higher than a certain confidence level. A set of features that are highly correlated with the target variable is obtained by keeping “important” features, which are statistically better than the best random features.

In this study, the Boruta package, retrieved from https://github.com/scikit-learn-contrib/boruta_py, is used to analyze the features mentioned in section “Feature construction.” Such package is performed with its default parameters.

### Feature ranking algorithms

Boruta is a feature selection algorithm that is designed to select features highly related to the classification. However, among them, some may be more important than others. Such task cannot be achieved by Boruta. Some other machine learning algorithms follow to further evaluate the importance of each selected feature. In this study, four feature selection methods [LASSO (Tibshirani, 1996), LightGBM (Ke et al., 2017), MCFS (Micha et al., 2008), and mRMR (Peng et al., 2005)] are employed. These methods can rank the features according to their importance to the classification.

#### Least absolute shrinkage and selection operator

Least absolute shrinkage and selection operator chooses important features that are beneficial to classification and eliminate those that are worthless or redundant (Tibshirani, 1996). This purpose is completed by building a linear regression model with L1 regularization. The coefficients of features can be a key indicator to suggest the importance of features. Features are ranked in a list in terms of their corresponding coefficients. Here, the LASSO package collected in Scikit-learn (Pedregosa et al., 2011) is used in this study. The obtained feature list is called LASSO feature list.

#### Light gradient boosting machine

A tree-based model can be used to evaluate the importance of features. LightGBM is a high-performance gradient boosting decision tree model that recurrently fits a new decision tree by using the negative gradient of the loss function of the current decision tree (Ke et al., 2017; Ding et al., 2022). LightGBM uses the total number of times each feature participates in creating tree nodes as a measurement of feature importance. Features are sorted in a list by the decreasing order of their above-mentioned times. Here, we use the LightGBM package in Python, downloaded from https://lightgbm.readthedocs.io/en/latest/, to analyze the features selected by Boruta. The list is called LightGBM feature list.

#### Monte Carlo feature selection

The MCFS method is a feature relevance estimation approach based on decision tree. This method is first introduced by Micha et al. and has been widely utilized in computational biology (Micha et al., 2008; Chen et al., 2018; Chen et al., 2019).

In this method, *s* feature groups are randomly constructed. For each feature group, a training dataset and a test dataset are randomly sampled from the original dataset. A decision tree is set up based on the training dataset and evaluated its performance on the test dataset. This procedure is repeated *t* times, thereby constructing *t* decision tree. After considering all *s* feature groups, a total of *t* × *s* decision trees are built. Based on these decision trees, a relative importance (RI) value is computed to assess the importance of each feature *g*, which can be expressed as follows:


(4) $$RI_{g}=\sum_{\tau =1}^{st}{\left(wAcc\right)}^{u}\sum_{n_{g}\left(\tau \right)}IG\left(n_{g}\left(\tau \right)\right){\left(\frac{no.inn_{g}\left(\tau \right)}{no.in\tau }\right)}^{v},$$

where *wAcc* is the weighted accuracy of the decision tree τ, *IG*(*n*_*g*_(τ)) represents the information gain of *n*_*g*_(τ), a DT node with the attribute *g* in tree τ, *no*.*inn*_*g*_(τ)stands for the number of samples in *n*_*g*_(τ), *no*.*in*τ stands for the number of samples in the tree root, and *u* and *v* are two settled positive integers. The present study uses the MCFS program retrieved from https://home.ipipan.waw.pl/m.draminski/mcfs.html. Default parameters are adopted. Based on decreasing order of RI values, features are ranked in a list, which is termed as MCFS feature list in this study.

#### Minimum redundancy maximum relevance

The mRMR evaluates the importance of features by both considering the relevance to the target variable and redundancies to other features (Peng et al., 2005; Wang et al., 2018; Zhao et al., 2018; Yu et al., 2020; Zhu et al., 2020; Chen et al., 2022). In theory, features with high relevance to the target variable and low redundancies to other features can receive high ranks in the final list. The relevance and redundancy are all evaluated by mutual information (MI). The ranks of features are determined by a loop procedure. In each round, the importance of one feature is assessed by the difference of its relevance to target variable and its redundancies to already-selected features. The feature with highest difference is selected and appended to the list. Here, the mRMR program obtained from http://home.penglab.com/proj/mRMR/ is used and is run with default parameters. The list generated by this method is called mRMR feature list.

As each of above-mentioned methods has its own merits and limitations, the usage of one method cannot fully uncover the essential biological functions related to uveitis. Each method can only depict a part of the whole picture on the biological functions of uveitis. By employing multiple methods, a more integrated picture can be obtained. Thus, we use all above-mentioned four feature selection methods, trying to mine biological functions of uveitis as complete as possible.

## Results

**FIGURE 1:**
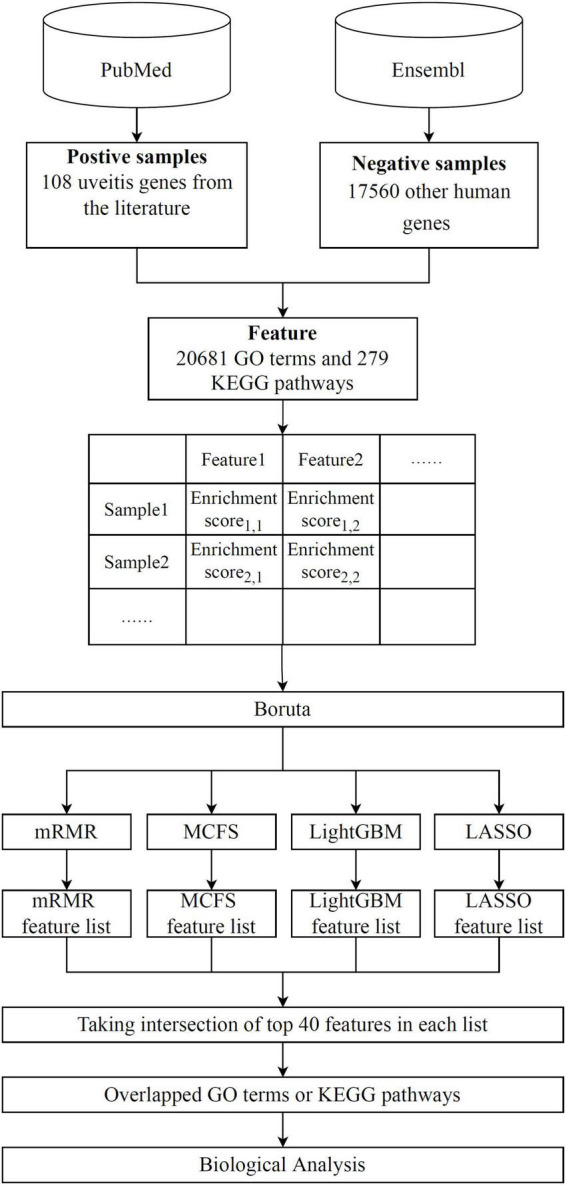
Flow chart of the whole analytical process. A total of 108 uveitis-associated genes and 17,560 other human genes are collected. Uveitis- and non-uveitis-related genes are then encoded by GO and KEGG enrichment scores, resulting in 20,681 GO term features and 297 KEGG pathway features. Boruta is used for feature filtering to obtain functional features related to uveitis. Subsequently, LASSO, LightGBM, MCFS, and mRMR are used to evaluate feature importance, and features are ranked from highest to lowest in terms of feature importance in four feature lists. Finally, highly relevant features were obtained by taking the intersection of the top 40 features in each feature list.

Advanced machine learning methods are used in this research, with the entire analysis procedure depicted in Figure 1. The results of each analysis stage are listed in detail below.

### Results of Boruta

The Boruta algorithm is performed on original features to eliminate nonrelevant features. Finally, 118 features related to the classification are chosen from 20,978 features, which are listed in Supplementary Table 1. These 118 features are functionally linked to the development of uveitis. Among these 118 features, 110 are related to GO terms, where the rest eight features are about KEGG pathways. Considering the features on GO terms are much more than those on KEGG pathways, such results are reasonable. For these 118 features, uveitis is highly correlated with some of them and weakly correlated with others, implying that the importance of the features should be investigated further.

### Results of feature ranking and evaluation

Four methods, namely, LASSO, LightGBM, MCFS, and mRMR, are used to measure the importance of 118 features selected by Boruta. A total of 118 features are ranked in four feature lists (LASSO, LightGBM, MCFS, and mRMR feature lists) based on their ability in distinguishing uveitis genes. These lists can be seen in Supplementary Table 1.

**FIGURE 2:**
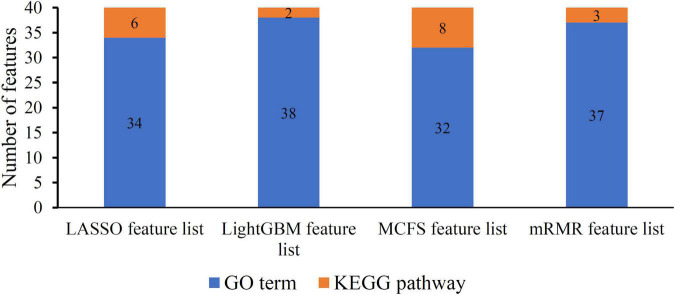
Distribution of top 40 features on GO terms and KEGG pathways in four feature lists. Features on GO terms are much more than those on KEGG pathways in each feature list.

**FIGURE 3:**
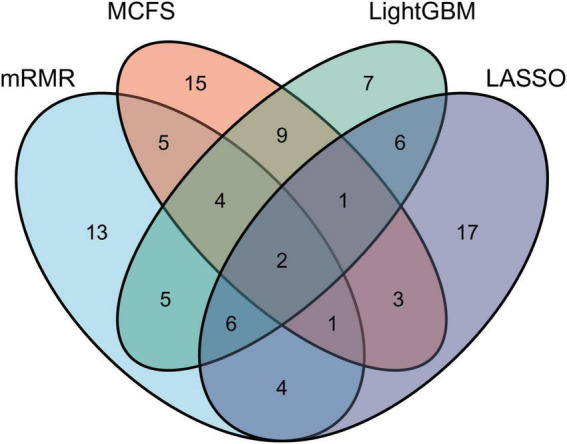
Venn diagram of top 40 features selected by LASSO, LightGBM, MCFS, and mRMR methods. Overlapping circles indicate the features identified by multiple methods. Two features are ranked high by all four feature ranking algorithms.

As mentioned in section “Feature ranking algorithms,” each method has its limitations. Some essential features related to uveitis can have high ranks in one feature list, whereas they may be underestimated in another feature list, that is, their ranks in the list are not high. Thus, an investigation on all feature lists is beneficial to fully uncover all essential biological processes of uveitis. For each feature list, top 40 features are picked up to comprise a feature set. Accordingly, four feature subsets are obtained. For each subset, the distribution on GO terms and KEGG pathways is illustrated in Figure 2, from which we can see that features on GO terms are much more than those on KEGG pathways in each feature set. Furthermore, a Venn diagram is plotted to display the intersection of these four sets, as shown in Figure 3. The detailed intersection results on these sets can be found in Supplementary Table 2. Two features appear in all feature sets, that is, they are highly ranked by all four methods. These features are considered to be most essential for distinguishing uveitis-related genes from other human genes, which are discussed in section “Functional features recognized by all methods.” As for the other features identified by three or less methods, they may also be important. Some of them are analyzed in sections “Functional features recognized by three methods,” “Functional features recognized by two methods,” and “Functional features recognized by only one method.”

## Discussion

On the basis of four commonly used machine learning methods, a set of functional features described by GO terms or KEGG pathways is identified to be associated with uveitis pathogenesis. As several features are predicted by at least two methods, the usage of multiple machine learning algorithms seems to be effective and robust for uveitis-associated functional feature selection. As mentioned in section “Results of feature ranking and evaluation,” we select the top 40 features from each list yielded by one machine learning method as features of interest. The typical features identified by all 4 methods, 3 of 4 methods, 2 of 4 methods, and by 1 method are picked up for detailed discussion.

### Functional features recognized by all methods

The first functional characteristic predicted by all methods is GO:0001841 describing neural tube formation. In 1983, researchers observed autoimmune-dependent attack on neural tubes during the pathogenesis of uveitis (Rahi and Addison, 1983), implying that neural tube formation is functionally associated with uveitis-related abnormal immune response. In recent years, an association analyses revealed that folic acid deficiency may contribute to the progression of uveitis by inducing neural tube defects (Sijilmassi, 2019), revealing the potential relationships between neural tube formation and uveitis. As for the next predicted shared feature, hsa04612, a KEGG pathway term, describes another immune-associated pathway, i.e., antigen processing and presentation in human beings. Antigen processing and presentation are confirmed to be related to uveitis by multiple publications from different perspectives (De Smet et al., 2001; Wakefield et al., 2016; Sharma and Jackson, 2017). Specifically, a specific subtype of HLA, i.e., HLA-B27, has been confirmed to participate in the pathogenesis of uveitis and associated with the susceptibility and prognosis of uveitis (Linssen and Meenken, 1995; Tay-Kearney et al., 1996; Wakefield et al., 2011). Therefore, the functional characteristics recognized by all methods have been validated to be functionally associated with uveitis, implying the efficacy and accuracy of our analysis.

### Functional features recognized by three methods

For functional characteristics predicted by three methods, GO:0043379 (memory T cell differentiation) has been predicted to be associated with uveitis. According to recent publications, memory T cell has been recognized during uveitis early in 2002 (Imai and Ohno, 2002), and a report in 2021 validates that memory helper T cells trigger the autoreactive immune response during uveitis (Egwuagu et al., 2021). Therefore, such predicted result has been validated by previous publications, implying the accuracy of our analysis. Another recognized GO term GO:0045625 (regulation of T-helper 1 cell differentiation) is also functionally associated with helper T cell-mediated immune responses, which can also be supported by two publications mentioned above. The next predicted term, i.e., GO:0071639, describes the positive regulation of monocyte chemotactic protein-1 production. Positive correlations between monocyte chemotactic protein-1 and pigment epithelium-derived factor, a key factor for uveitis (Zipplies et al., 2009), have been observed (Yoshida et al., 2007). Therefore, as an immune related regulator, monocyte chemotactic protein-1 is a potential biomarker for uveitis.

### Functional features recognized by two methods

The first GO term predicted by two methods is GO:0046642, which describes the negative regulation of alpha–beta T cell proliferation. Few publications compared the roles of alpha–beta and gamma–delta T cells during uveitis. Only one mouse model-based uveitis study confirmed that at least in a mouse model, alpha–beta T cells are directly associated with autoimmune-triggered uveitis, validating the efficacy and accuracy of our analysis. Another identified GO term by two methods is GO:0045078 (generally described with GO:0032729), which describes the positive regulation of interferon-gamma production. Interferon-gamma has been shown to be associated with tuberculous-initiated uveitis (Ang et al., 2014). *Staphylococcus aureus* infection described by hsa05150 has also been predicted to be associated with uveitis. *S. aureus* as a Gram-positive bacteria has been reported to be identified in various patients with uveitis and has been shown to initiate uveitis-associated autoimmune responses (Rosenbaum et al., 1980; Lin, 2015). Therefore, such bacterial infection is associated with uveitis.

### Functional features recognized by only one method

Some characteristics that have only been predicted by one method remain. GO:0042102 describes the positive regulation of T cell proliferation. As discussed above, T cell-associated biological processes are shown to be related to uveitis. Therefore, T cell proliferation is speculated to be associated with uveitis without direct supports. The next predicted GO term is GO:0005151, which describes interleukin-1 type II receptor binding. Interleukin-1 alpha, interleukin-1 beta, and tumor necrosis factor are shown to be associated with endotoxin-induced uveitis (Yoshida et al., 1994; Tsai et al., 2009). Chemokine activity (GO:0008009) has also been identified by one machine learning method. Different from T cell-mediated immune responses, which can be found in almost every uveitis case, chemokine abnormality has been commonly observed in acute active uveitis (Verma et al., 1997; Van Kooij et al., 2006), indicating that such biological function may be associated with the rapid progression of uveitis.

Overall, as discussed above, some top features identified by different machine learning methods have been validated to participate in the initiation and progression of uveitis, implying that machine learning methods are effective tools in recognizing disease-associated biological functions. Among these methods, LightGBM has more features that are also identified by other methods (Figure 3), implying that LightGBM may be the most proper method for investigating uveitis. Therefore, our study recognizes a series of functional characteristics (GO: biological process, cellular components, and molecular function and KEGG: pathways) associated with uveitis and provides a new approach for disease mechanism exploration.

## Conclusion

In this study, a computational analysis, incorporating multiple feature selection methods is developed to extract essential functional terms (GO terms and KEGG pathways) that can be used to distinguish genes associated with uveitis. First, we collect 108 genes associated with uveitis from previous literature as positive samples and further collect 17,560 other human genes as negative samples. Subsequently, GO and KEGG enrichment scores are used to encode uveitis- and non-uveitis-related genes, yielding 20,681 GO term features and 297 KEGG pathway features. Finally, we combine Boruta and four feature ranking algorithms (LASSO, LightGBM, MCFS, and mRMR), to rank the features by importance and obtain features that are highly correlated with uveitis. We elucidate the association of these features with the occurrence and development of uveitis through the literature. For example, GO:0001841, which describes neural tube formation, is recognized by all methods and is functionally associated with uveitis-related abnormal immune response. In summary, this study has used GO terms and KEGG pathways to characterize uveitis genes at the functional level.

## Data availability statement

Publicly available datasets were analyzed in this study. This data can be found here: Lu et al. (2017).

## Author contributions

SL and JZ designed the study. SL and HW performed the experiments. SL, HW, and JZ analyzed the results. HW wrote the manuscript. All authors contributed to the research and reviewed the manuscript.

## Footnotes

1
https://pubmed.ncbi.nlm.nih.gov/

## Supplementary material

Supplementary Table 1 A total of 118 features are obtained after Boruta filtering and they are ranked by LASSO, LightGBM, MCFS, and mRMR.

Supplementary Table 2 Venn results for the top forty features in four feature lists ranked by LASSO, LightGBM, MCFS, and mRMR.
